# The Impact of the Covid-19 Pandemic on the Well-Being and Diabetes Management of Adolescents With Type 1 Diabetes and Their Caregivers: A Scoping Review

**DOI:** 10.3389/fcdhc.2022.835598

**Published:** 2022-03-08

**Authors:** Giulia Bassi, Elisa Mancinelli, Gaia Dell’Arciprete, Silvia Salcuni

**Affiliations:** ^1^Department of Developmental and Socialization Psychology, University of Padova, Padova, Italy; ^2^Digital Health Lab, Centre for Digital Health and Wellbeing, Fondazione Bruno Kessler, Trento, Italy

**Keywords:** covid-19, type 1 diabetes, adolescents, caregivers, well-being, scoping review

## Abstract

The Covid-19 pandemic and its related restriction measures might negatively impact diabetes management and well-being of adolescents with Type 1 Diabetes Mellitus (T1DM) and their caregivers. Accordingly, the present scoping review is aimed at mapping the literature in line with the question *“How has the Covid-19 influenced diabetes management and well-being of adolescents with T1DM and their caregivers?”*. A systematic search has been conducted through three academic databases. Studies carried out during the Covid-19 pandemic focused on adolescents aged between 10 and 19 years with T1DM and/or their caregivers were included. A total of 9 studies, performed between 2020 and 2021, have been identified. In particular, N = 305 adolescents with T1DM and N = 574 caregivers were considered. Overall, studies were not specific in reporting adolescents’ age, and only 2 studies were primarily focused on the adolescent population with T1DM. In addition, studies were mainly focused on evaluating adolescents’ glycemic control, which remained stable or has improved throughout the pandemic. Contrarily, psychosocial variables have been marginally considered. Indeed, only one study investigated adolescents’ diabetes distress, which remained stable from pre-to during post-lockdown, albeit improving among girls, specifically. As regards caregivers of adolescents with T1DM, studies showed mixed results concerning their psychological state during the Covid-19 pandemic. Prevention measures, which are aimed at supporting adolescents with T1DM during the lockdown, were considered by one study only, showing the favorable role of telemedicine during lockdown for adolescents’ glycemic control. Altogether, the current scoping review has identified many shortcomings of the available literature, which are given by the limited specificity of the age group considered and by the limited consideration of psychosocial variables, particularly their interplay with the medical ones.

## Introduction

Covid-19, the respiratory illness caused by the SARS-CoV-2 virus, was declared a global pandemic in March 2020 by the World Health Organization (1) due to its extremely contagious nature and associated high mortality rates. Although pediatric patients with Type 1 Diabetes Mellitus (T1DM) seem not to be at an increased risk of morbidity and mortality for Covid-19 compared to adults (2), they still are deemed as a particularly vulnerable population, especially considering that DM was found to be one of the most frequent comorbidities among people who got infected with Covid-19 (3). When it comes to diabetes management, which consists of the day-to-day actions required from patients with DM to consistently keep their disease under control, such as undertaking daily insulin shots, regularly monitoring their blood glucose, and maintaining a healthy lifestyle by being physically active and following a balanced dietary regimen (4, 5), adolescents already represent an at-risk group. Indeed, on top of having to deal with the multiple biopsychosocial changes (6) typical of this developmental phase, they are also required to take on a greater sense of responsibility for their daily diabetes management (7).

Before the Covid-19 pandemic, plenty of evidence had already been gathered on how T1DM is a challenging condition for adolescents to live with: its management is often regarded as tedious, complex and time-consuming, and may potentially lead to the development of mental health issues (8, 9). More specifically, Rechenberg and colleagues (8) highlighted how adolescents with T1DM experience heightened levels of anxiety symptoms regarding both the disease itself and diabetes-management specific tasks; other recent studies also confirmed the high prevalence of depression and anxiety symptoms in youth with T1DM and pointed out how these can lead to poorer diabetes management and glycemic control (10, 11).

Within this context, the recent outbreak of the Covid-19 pandemic may become an additional risk factor for adolescents with T1DM. Indeed, restrictions put in place to contain the spread of Covid-19 have implied major repercussions in terms of access to the healthcare system and its resources (12, 13), hindering the possibility for patients to keep up with their follow-up visits with the healthcare professionals. In addition, the lockdown measures imposed to contain the spread of the virus have led to drastic and abrupt changes in adolescents’ lifestyles, which could be a further risk factor considering that a consistent daily routine, physical activity, and a healthy diet are pivotal in enabling more efficacious diabetes management among adolescents (14). Nonetheless, the greater closeness between adolescents and their families imposed by the lockdown might be regarded as a favorable aspect for diabetes management, since previous studies have shown that increased and constant family involvement within adolescents’ diabetes management was predictive of better blood glucose monitoring and glycemic control (15). However, existing literature highlighted how parents of a child with chronic disease are themselves at increased risk for mental health issues: the burden of partaking in their offspring’s everyday disease management can determine high levels of stress, anxiety, and depression symptoms (16–18). Recent literature (19) also shows how parents’ and their child’s well-being are strictly intertwined: as mentioned above, adolescents’ DM often has a detrimental effect on their parents’ well-being; this, on its part, can reflect in low parental self-efficacy, which in turn was shown to have negative effects on their child’s self-management and metabolic control (19). On the other hand, there is evidence that adolescents with strong family support display better adherence and glycemic control (20). This, overall, illustrates how both parents and their child with T1DM are involved in maintaining positive diabetes management, which is therefore deemed as a “family disease” (21, 22).

The aforementioned evidence highlights the need to investigate how the ongoing pandemic has affected and still affects adolescents with T1DM, verifying how this topic has been explored within the literature in order to collect evidence and suggestions on which to base and tailor future research and interventions. Therefore, the current scoping review was conducted to map the relevant literature related to the impact of the Covid-19 pandemic on adolescents with T1DM and their caregivers. The broader question guiding this scoping review is *“How has the Covid-19 influenced diabetes management and well-being of adolescents with T1DM and their caregivers?”*. In this regard, it should be noted that the present study followed the definition of adolescence proposed by WHO, according to which it is a period that covers the age from 10 to 19 years (23). Three different and more specific questions were further outlined under this umbrella:

1. What are the protective and risk factors among adolescents with T1DM in the context of Covid-19?
2. How did Covid-19 influence the role of caregivers in supporting their adolescents with T1DM?
3. Which prevention measures have been implemented so far to support adolescents with T1DM in the context of Covid-19?

## Materials and Methods

### Scoping Review

Scoping reviews are aimed at mapping the core concepts that underlie a precise research topic by analyzing its nature, features as well as the amount of investigation conducted (24), while providing a global overview of the content and/or findings of it. Indeed, scoping reviews are commonly applied “for reconnaissance” (22, p. 141), that is, to allow for clarification when a body of literature has not yet been reviewed or when there may be inconsistencies and/or heterogeneity on a topic (25).

### Search Strategy

**Figure 1:**
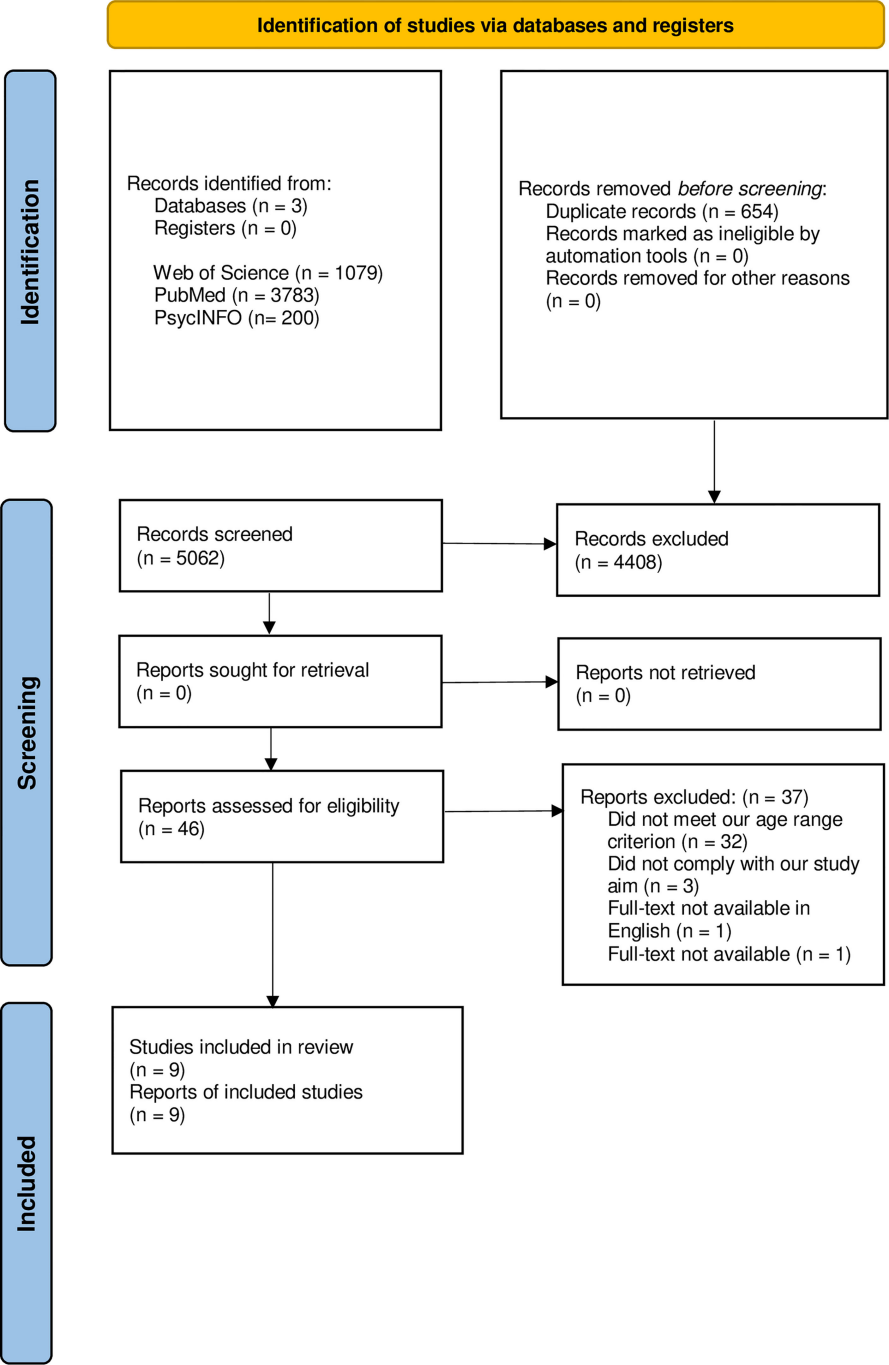
The PRISMA-ScR 2020 flow diagram of the literature search and the selection process (26).

The current scoping review was conducted in compliance with the Preferred Reporting Items for Systematic Reviews and Meta-Analysis (PRISMA) extension for Scoping Reviews (PRISMA-ScR) guidelines (26), as shown in **Figure 1**.

Three electronic bibliographic databases, specifically Web of Science, PubMed and PsycINFO, were screened to identify studies published during the years 2020 and 2021, to ensure they were conducted during the Covid-19 pandemic. Studies were identified using the following MeSH terms: type 1 diabetes mellitus, adolescent* OR adolescence, caregiver* OR parent*, well-being, anxiety, stress, depression, health-related quality of life, quality of life, COVID-19, diabetes management.

### Study Screening

The titles and abstracts retrieved from the above-mentioned electronic databases were independently screened by one author (GDA) and any doubt has been solved by consulting two other authors (GB and EM). This initial screening has been conducted by a single author because of its preliminary nature. The more thorough full-text screening needed to assess studies eligibility, in line with inclusion and exclusion criteria, has been performed independently by two authors (GB and EM) through a double-blind process; any doubt or conflict has been solved by consulting a third author (SS).

### Inclusion Criteria

Studies were included in the present study if they fulfilled the following inclusion criteria: (I) focused on adolescents with T1DM aged 10 to 19 years [according to the WHO’s definition of adolescence (23)]; (II) focused on the caregivers of adolescents with T1DM aged 10 to 19 years; (III) being published in academic journals during the Covid-19 pandemic and (IV) having undergone peer-reviewing; being written in English; (V) investigated the impact of the Covid-19 pandemic upon adolescents and/or caregivers’ regarding diabetes management, well-being, depression, anxiety, stress, diabetes distress, quality of life and adolescents’ health-related quality of life, and/or (VI) evaluated prevention measures deployed during the Covid-19 pandemic to support adolescents with T1DM both medically and psychologically.

### Exclusion Criteria

Studies were excluded if they met the following criteria: (I) being a review, dissertation, conference abstract, editorial, or commentary; (II) focused solely on children, young adults, adults, or adolescents with Diabetes Ketoacidosis (DKA); (III) provided only aggregated data for children and adolescents or adults; (IV) focused on any other medical disease different from T1DM.

### Data Extraction and Management

Data extraction was independently performed by two authors (GB and GDA) and any disagreement was resolved by consulting a third author (EM). The data extracted were the studies’ DOI, first author’s surname, year of publication, country of origin, study design, period (date and month) of the Covid-19 pandemic in which the study was conducted, adolescents and/or caregivers’ characteristics (e.g., sample size, age, gender), diabetes duration, assessment instruments used to investigate the mentioned variables and, lastly, outcomes of interest (i.e., adolescents and/or caregivers diabetes management, well-being, depression, anxiety, stress, diabetes distress, quality of life, adolescents’ health-related quality of life and prevention measures divulged to adolescents with T1DM).

## Results

### Search Process Results

The search process results are shown in **Figure 1**. A total of 5062 studies were identified following the initial database search. After duplicate removal, the preliminary screening of titles and abstracts was performed on a total of 4408 studies, resulting in 46 studies, which were further evaluated for eligibility through full-text screening. In line with the inclusion and exclusion criteria, 9 studies were included, and 37 studies were excluded; the full list of reasons for exclusion is reported within the supplementary material (**Supplementary Material Table S1**).

### Studies’ Characteristics

Characteristics of the included studies are summarized in **Tables 1A****,** **B**. Studies were published between 2020 and 2021 and were all conducted in 2020, thus during the first wave of the Covid-19 pandemic, including the lockdown period. A total of N = 305 adolescents (50.07% female) and N = 574 caregivers (95.45% female) were identified.

**Table 1A:** Studies Characteristics–Population.

Author, year	Country	Study Design	Population [N, Gender, Age (*M ± SD, Range)*]		Socio-demographic characteristics [Ethnicity, SES]	Diabetes duration (years)
			Caregivers	Adolescents		
Alessi et al. (27)	Brazil	Web-based qualitative research	▪ N = 144	▪ N = N.R. ▪ Age = 11.7 ± 4.3	N.R.	N.R.
Alessi et al. (28)	Brazil	Cross-sectional study	▪ N = 381 ▪ (95.3% female 89.8% mothers) ▪ Age = 40.3 ± 8.0	▪ N = 146	▪ 68.8% white ▪ 54.1% lower-middle income	N.R.
Brener et al. (29)	Israel	Longitudinal study	N.A.	N.R.	N.R.	N.R.
Ceconi et al. (30)	Italy	Longitudinal study	N.A.	▪ N =13 (62% female) ▪ Age = 14.2 ± 3	N.R.	N.R.
Di Dalmazi et al. (31)	Italy	Longitudinal study	N.A.	▪ N = 24 (37.5% female) ▪ Age = 13-17 ▪ Median age: 15.6 [IQR: 14.2-16.8]	N.R.	▪ Median age = 7.2 ▪ [IQR = 5.1-9.5]
Elhenawy et al. (32)	Egypt	Cross-sectional study	N.A.	▪ N =61 (57.4 female) ▪ Age: 10-18 years	N.R.	▪ 6.6% (6 months-1 year) ▪ 32.8% (1-5 years) ▪ 60.7% (>5 years)
Mianowska et al. (33)	Poland	Longitudinal study	▪ N = 56 (81.8% mothers) ▪ Age = N.R.	▪ N = 55 (63.6% male) ▪ Age = 12-18 ▪ Median age: 14.4 ▪ [IQR = 13.6-16.1]	N.R.	▪ Median age = 4.5 ▪ [IQR = 1.8-8.8]
Minuto et al. (34)	Italy	Longitudinal study	N.A.	▪ N = 58 ▪ Age = 14-18	N.R.	N.R.
Telford et al. (35)	New Zealand	Cross-sectional study	N.R.	▪ N = 33 (58% female) ▪ Age = 14.1 ± 1.6 (11-18)	▪ N = 26 European ▪ N = 3 Māori ▪ N = 3 Pacific ▪ N = 1 Other ▪ NZDep18 = 4.7 ± 3.1 ▪ School decile = 6.2 ± 3.2	6.2 ± 3.3

NZDep18, New Zealand Deprivation Index 2018; IQR, Interquartile Range; School decile, “School decile is calculated by the Ministry of Education and reflects the proportion of students within the school roll who come from low socioeconomic neighborhoods. Deciles are denoted on a scale from 1–10. where lower decile represents lower income communities.”(9. p. 3).

N. R., not reported; N. A., not available.

**Table 1B:** Studies Characteristics–Outcome(s) of interest.

Author, year	Assessment period	Outcome	Assessment tool
Alessi et al. (27)	May 18^th^-June 9^th^, 2020	▪ Burden of care ▪ Personal emotional impact	Qualitative interview
Alessi et al. (28)	May 18^th^-June 9^th^, 2020	▪ Pandemic-related emotional burden ▪ Mental disorders ▪ Diabetes-specific emotional burden	▪ *Ad hoc* 5-points Likert scale questionnaire. ▪ Self-Report Questionnaire (SRQ-20). ▪ *Ad hoc* 5-points Likert scale self-report.
Brener et al. (29)	▪ Pre-lockdown = February 23^rd^-March 7^th^, 2020 ▪ During lockdown = March 25^th^-April 7^th^, 2020	Glycemic control	CGM metrics (TIR, HbA1c, mean glucose, glucose SD) assessed through ambulatory glucose profile report.
Ceconi et al. (30)	▪ Pre-lockdown = February 10^th^-23^rd^, 2020 ▪ During lockdown = March 9^th^-22^nd^, 2020 ▪ Semi-lockdown = May 4^th^-17^th^, 2020 ▪ Post-lockdown = May 18^th^-31^st^, 2020	Glycemic control	CGM metric (TIR, TAR, TBR) and GMI, assessed through tele-visit reports.
Di Dalmazi et al. (31)	▪ Pre-pandemic = January 30^th^-February 19^th^, 2020 ▪ Pre-lockdown = February 20^th^-March 10^th^, 2020 ▪ During lockdown = March 11^th^-20^th^, 2020	▪ Glycemic control ▪ Physical activity	▪ CGM metrics (TIR, TAR, TBR, Mean glucose, glucose SD, LBGI, HBGI), GMI, HbA1c. ▪ International Physical Activity Questionnaire-Short Form (IPAQ-SF).
Elhenawy et al. (32)	▪ Pre-lockdown ▪ Post-lockdown	Diabetes management Glycemic control	▪ Questionnaire administered online, through phone calls. ▪ HbA1c.
Mianowska et al. (33)	▪ Pre-pandemic = November 2019-February 2020 ▪ During lockdown = April 2020	▪ Diabetes distress adolescents ▪ Diabetes distress caregivers ▪ Glycemic control	▪ Problems Areas in Diabetes-Teen (PAID-T). ▪ Problem Areas in Diabetes-Parents of Teens (P-PAID-T). ▪ HbA1c (CGM, SMBG)
Minuto et al. (34)	▪ Pre-pandemic = February 10^th^-23^rd^, 2020 ▪ During lockdown = April 17^th^-30^th^, 2020	▪ Glycemic control ▪ Physical activity	▪ TIR, TAR, TBR, HbA1c. ▪ Sport in hours/week.
Telford et al. (35)	During lockdown = May 2020	▪ Glycemic control ▪ Physical activity	▪ HbA1c ▪ Physical Activity Questionnaire for Older Children (PAQ-C) aged 11-12 years; Physical Activity Questionnaire for Adolescents (PAQ-A) aged 12-18 years; Children’s Physical Activity Questionnaire (CPAQ; parent-proxy measure)

CGM, Continuous Glucose Monitoring; GMI, Glucose monitoring Index; LBGI, Low Blood Glucose Index; HBGI, High Blood Glucose Index; SMBG, Self-monitoring of blood glucose; TIR, Time in Range; TAR, Time Above Range; TBR, Time Below Range.

Among the included studies, N = 2 (30, 35) were specifically focused on adolescents, N = 4 (29, 32–34) compared children and adolescents, N = 1 (31) compared children, adolescents, and adults, and N = 2 (27, 28) compared caregivers of children with T1DM *vs.* caregivers of adolescents with T1DM. In this regard, it should be noted that high heterogeneity has emerged within studies’ reporting of adolescents’ age: only one study (35) provided mean, standard deviation (SD) and specific age range of participants, one study (30) provided mean age and SD, yet not the age range, and one (29), albeit providing this information for the aggregated sample comprising both children and adolescents, did not mention the specific demographic characteristics of the adolescent subsample. About the remaining included studies, only N = 2 reported the sample’s age range (32, 34), while N = 2 (31, 33) reported the sample’s median age, IQR and age range. Furthermore, the two studies, which focused on caregivers (27, 28), did not report information regarding their child’s age. Lastly, information on the household’s socio-economic status was reported by N = 2 studies only (29, 35).

**Figure 2:**
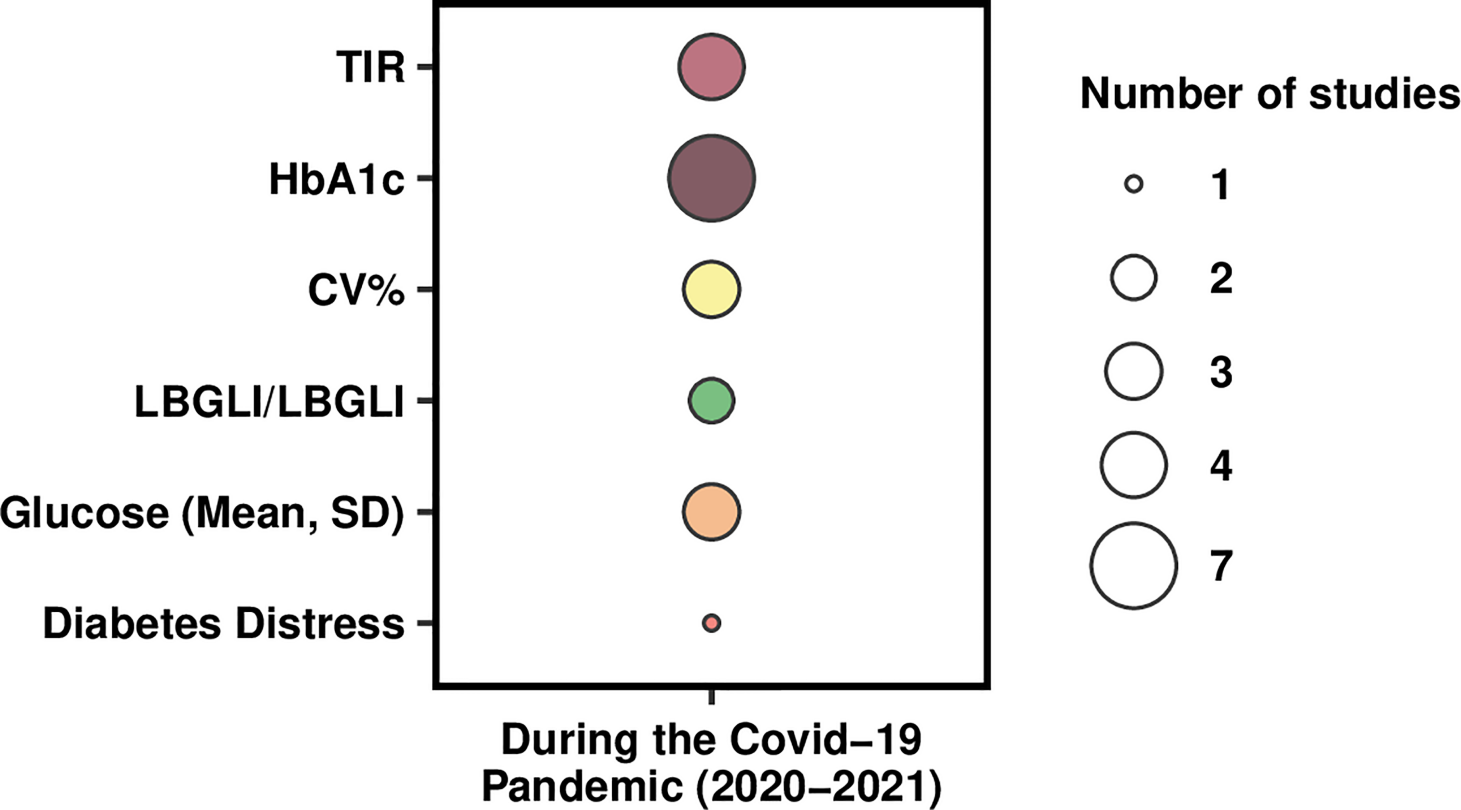
Bubble plot showing the distribution of identified outcomes.

Overall, N = 5 studies focused solely on the medical components of adolescents’ T1DM, thereby analyzing their glycemic control as assessed through the glycemic indices shown in **Figure 2**. More specifically, N = 4 studies (29–31, 34) reported the Time in Range (TIR), comprising also the Time Above Range (TAR) and the Time Below Range (TBR) which were reported by N = 3 (30, 31, 34) studies. Hemoglobin A1c (HbA1c) was instead reported by N=6 studies (29, 31–35), with N = 2 studies also reporting information about the Glucose Management Indicator (GMI) (30, 31) representing the estimates A1c (eA1c). The Low and High Blood Glucose Indices (LBGI and HBGI, respectively) were reported by N = 2 studies (29, 31), while the Mean Glucose by N = 3 studies (29, 31, 34); of these, N = 2 (29, 31) also provided the sample’s glucose SD. The coefficient of variation expressed in percentage (CV%) was reported by N = 3 studies (29, 30, 34). Furthermore, among these studies, N = 3 (29, 30, 34) only evaluated and provided information on medical measures, while N = 2 (31, 35) provided both medical measures and physical activity-related aspects. Remarkably, only one study (33) (see **Figure 2**) took into account adolescents’ psychological variables by investigating their diabetes distress (DD).

Both studies, which focused on caregivers (27, 28), evaluated their emotional burden and worries referred to their child’s diabetes management. One also evaluated caregivers’ likelihood of developing mental health issues during lockdown (28). It is noteworthy that one study (32) evaluated perceived stress, but unclearly reported if it was assessed referring to adolescents with T1DM or to their caregivers.

### Results of Individual Evidence and Overall Synthesis

The results of the included studies are charted in tabular form (**Tables 2A****–****C**) in line with the research questions posed by the current scoping review.

**Table 2A:** RQ1: What are the protective and risk factors among adolescents with T1DM in the context of Covid-19?

Author	Protective Factors	Risk Factors	Overall results	Relevant study limits
Brener et al. (29)	▪ Being an older adolescent was associated with improved TIR, lower glucose SD, and reduced CV.	▪ A lower socioeconomic position was associated with increased delta-mean glucose.	▪ Glucose variability was more stable among adolescents compared to children, both before and during lockdown.▪ Adolescents, compared to children, showed improved variability markers during lockdown.	▪ Only considered patients that used CGM*.
Di Dalmazi et al. (31)	_	_	▪ Mean daily time spent sitting was higher among adolescents compared to children. ▪ Among adolescents, all CGM measures remained unchanged from before the lockdown period, while they showed improvement among children. ▪ Glucose SD among adolescents decreased from before to during the lockdown period.	▪ Protective factors were solely evaluated among adults. ▪ Only considered patients that used CGM*. ▪ Compared to the broader literature, the adolescent group had better mean HbA1c levels*.
Elhenawy et al. (32)	_	_	▪ Adolescents (and children aged 5-10 years) showed increased HbA1c levels from pre-lockdown to after lockdown, compared to children aged 0-5 years. ▪ Adolescents (and children aged 5-10 years) showed worse diabetes control during lockdown compared to children aged 0-5 years.	▪ Protective/risk factors were solely considered referring to the aggregated sample (i.e., youth aged 0-18). ▪ Unclear if perceived stress was assessed on youth or their caregivers.	
Mianowska et al. (33)	▪ During lockdown, being female was associated with significantly decreased DD.	▪ High adolescents’ DD before the pandemic was associated with reduced changes in DD scores during the lockdown.	▪ Before the pandemic, adolescents’ and their caregivers’ DD positively associated with adolescents’ BMI. ▪ Adolescents showed reduced DD during lockdown, especially girls. ▪ Females presented significantly greater DD than boys before the pandemic. ▪ DD remained unchanged among male adolescents from before to during lockdown.	▪ DD assessment during lockdown was performed by phone*.	
Minuto et al. (34)	_	_	▪ Adolescents showed improved glycemic control from pre- to post-lockdown. ▪ Significant reduction of physical activity from pre- to post-lockdown among all age groups (i.e., children, adolescents, adults).	▪ The protective role played by physical activity that emerged was solely evaluated considering the aggregated sample.	
Telford et al. (35)	_	▪ Having T1DM predicted increased BMI during lockdown.▪ Belonging to a lower socio-economic position and higher BMI is associated with poorer glycemic control.	▪ Adolescents with T1DM showed significantly higher BMI compared to the control group. ▪ Physical activity level showed no association with adolescents’ socioeconomic position, HbA1c levels, or the method of insulin delivery. ▪ BMI was negatively associated with physical activity during lockdown. ▪ All participants showed reduced physical activity during lockdown; rates did not differ between the T1DM and control group, nor between males and females.	▪ No information about pre-lockdown physical activity levels*.	

BMI, Body Mass Index, CGM: Continuous Glucose Monitoring; CV, Coefficient of Variation; DD, Diabetes distress; glucose SD, glucose standard deviation; TIR, Time in range; *Authors’ reported study limit relevant to the current scoping review.

**Table 2B:** RQ2: How did Covid-19 influence the role of caregivers in supporting their adolescents with T1DM?

Author	Constructs	Psychological consequences	Diabetes-management related consequences	Relevant study limits
Alessi et al. (27)	▪ Burden of care ▪ Personal emotional impact	▪ The burden of care in terms of lack of information on the association between COVID infection and T1DM. ▪ Reported exhaustion and weariness toward the economic situation and health institutions’ sanitary conditions. ▪ 1/3 reported exhaustion and overload consequent to assume the double role of full-time caregiver and family provider. ▪ Distress referred to the perceived ineptitude resulting from difficulties in balancing work and caregiving demands. ▪ Reported feelings of overwhelm and lack of privacy.	▪ Anxiety, uncertainty, and feelings of hopelessness resulting from suspension of medical appointments. ▪ Frustration has given by difficulties linked to glycemic control. ▪ Guilt and uncertainty about ensuring proper nutritional food because of financial difficulties due to the COVID-19 situation.	▪ Interviews did not allow discussion*. ▪ The majority of the sample (95.6%) was female*.
Alessi et al. (28)	▪ Pandemic-related emotional burden ▪ Mental health linked to social distancing ▪ Diabetes-specific emotional burden	▪ No difference between caregivers of youth with T1DM and caregivers in the control group in terms of overall relationships satisfaction and family perceived welcomeness ▪ No difference between caregivers of youth over 12 with T1DM and caregivers in the control group in the likelihood of developing mental health issues. ▪ Increased likelihood of showing child-related concern, personal emotional burden, and child-related emotional burden among caregivers of youth over 12 and with T1DM.	▪ Increased difficulty in accessing medical assistance and purchasing medical supplies among parents of youth with T1DM aged both below and above 12 during the pandemic.	▪ The majority of the sample (95.3%) was female*. ▪ The control group was recruited through “medical student leagues”*.
Mianowska et al. (33)	▪ Diabetes distress	▪ Caregivers of adolescents with T1DM showing high DD levels before lockdown reported a reduced change in DD scores during lockdown. ▪ Overall, caregivers showed no difference in DD levels from pre- to during lockdown.	▪ Type of glucose monitoring and changing the monitoring tool did not influence the DD levels of caregivers of adolescents with T1DM.	▪ DD assessment during lockdown was performed by phone*. ▪ No socio-demographic information of caregivers. ▪ Caregivers were for the majority (81.8%) mothers.

DD, Diabetes distress; *Authors’ reported study limit relevant to the current systematic review.

**Table 2C:** RQ3: Which prevention measures have been implemented so far to support adolescents with T1DM in the context of Covid-19?

Author	Prevention Measure	Characteristics	Results	Relevant study limits
Ceconi et al. (30)	Health care continuous assistance through telemedicine	N/R	Telemedicine conducted during the lockdown period and the month following the relaxation of restrictions favored stable and improved glycemic control among adolescents with T1DM. Hypothesized “dragging effect” of improved glycemic control following lockdown as consequent to constant telemedicine interactions with the physician.	Not possible to assess telemedicine efficacy on glycemic control (e.g., no control group)*. Insufficient information on the procedure and overall unclear methodology. Did not evaluate contextual variables (e.g., socio-demographic information). No information on the analysis was performed.

*Authors’ reported study limit relevant to the current scoping review.

N. R., not reported.

Many inconsistencies and overall heterogeneity in information reporting have emerged, in particular regarding adolescents’ age. Notwithstanding this, for what concerns diabetes management, included studies showed a stable and even improved glycemic control among adolescents with T1DM throughout the pandemic. Notably, a study has reported stable TIR and HbA1c values from pre-lockdown (31), while others have shown improved TIR between the two periods (29, 30) as well as improved TIR (30, 34) and HbA1c (34) from pre-and post-lockdown. However, one study (32) reported a worsening in glycemic control as indexed by increased HbA1c from pre-to post-lockdown. None of the included studies has investigated differences in metabolic control based either on the type of insulin therapy regimen or the type of glucose monitoring.

The protective or risk factors for adolescents’ adjustment throughout the pandemic have not been specifically evaluated by the included studies, as they have mainly focused on providing a descriptive “overview” of the situation by mostly referring to the medical aspect of T1DM. Nonetheless, the need to take action as regards the much-reduced physical activity during the lockdown period was reported (34, 35), particularly in light of a foreseen continuation of pandemic-related restrictions. Despite this, some authors (30, 31, 34) have developed a “positive lockdown effect” hypothesis, whereby more stable and constant life routines and reduced everyday life stress might have accounted for the above-reported stable or improved glycemic control shown by adolescents with T1DM. It is noteworthy that the psychological impact of both the pandemic and lockdown has been investigated by one study only (33), which has compared adolescents’ DD levels pre-and during the lockdown, showing a decrease between these two periods, especially in girls. In this regard, the authors (33) pointed out that before the Covid-19 pandemic neither adolescents’ nor their parents’ DD was influenced by their mode of insulin therapy (i.e., multiple daily injections *vs.* continuous subcutaneous insulin infusion) nor by the type of glucose monitoring [continuous glucose monitoring (CGM) *vs.* self-monitoring of blood glucose (SMBG)]. Comparably, during the lockdown period, they observed no changes in DD levels neither among adolescents who had changed the type of glucose monitoring (from SMBG to CGM) nor among those continuing with SMBG.

In line with the scarce investigation of adolescents’ psychological adjustment, no specific prevention measures seem to have been deployed and evaluated during the pandemic to specifically support the overall adjustment of adolescents with T1DM. However, Ceconi and colleagues (30), stressed the potentiality of telemedicine to support adherence and self-efficacy during the lockdown.

The impact of the pandemic upon caregivers was mainly reflected in increased perceived burden in both diabetes management and child-related worry and burden (27, 28). Still, results also showed that caregivers of adolescents with T1DM did not significantly differ from parents in general in the likelihood of presenting mental health issues during a lockdown.

## Discussion

The present scoping review is aimed at evaluating evidence useful to answer the previously reported three questions that fall within the broader question “*How has Covid-19 influenced diabetes management and well-being among adolescents with T1DM and their caregivers?”*. To reach this goal, five thousand sixty-two titles were initially identified; yet only nine studies were eligible to be included. Notably, during the full-text screening, several studies were removed since their attention was directed towards children with T1DM under or equal to 9 years of age. Indeed, the adolescent group with T1DM was scarcely taken into consideration within the literature, and the studies who did often showed results aggregated with those of children under the age of 9 (see **Supplementary Material**): among the included studies, only 23% (two studies) were specifically oriented towards the effect of Covid-19 on adolescents with T1DM (30, 35).

Overall, the included studies have depicted the general *scenario* of the effect of Covid-19 among adolescents with T1DM and their caregivers: this lays the basis for the development of future research, which, beyond the reported choice of age group to investigate, should also be more careful and comprehensive in defining the outcomes to investigate. On this matter, the majority of the included studies focused on evaluating the impact of Covid-19 on medical indices among adolescents with T1DM. In general, they underlined a stable and even improved TIR and HbA1c pre-, during and post-lockdown (29–31, 34), except for one study that showed a downward trend of glycemic control pre-and post-lockdown (32). Another relevant result that emerged was the reduction in physical activity, which reflects the fact that the lockdown period has led to a more sedentary lifestyle (34, 35). In this scenario, only one study evaluated the effect of Covid-19 on DD among adolescents with T1DM, thereby taking into account the psychosocial side. Authors pointed out that adolescents experienced moderate DD levels, which although decreased only among girls from pre-to-during lockdown (33). Nonetheless, before the Covid-19 pandemic, girls showed significantly higher DD than boys (33), which suggests that the more stable life routine allowed by lockdown, as reported by other authors (27, 30, 34) referring to its effect upon glycemic control, was much needed and useful for female adolescents as regards their DD. Referring, instead, to diabetes management and glycemic control, evidence suggested that being older adolescents favored improved TIR and reduced glucose variability expressed both as the glucose SD and the CV% throughout the Covid-19 pandemic. In addition to their greater maturity, the increased detachment from the family unit and the increased sense of responsibility that can be observed during late adolescence might further push adolescents toward a greater and more autonomous diabetes management, which in turn leads to improved glycemic control. Contrarily, lower socio-economic status and higher BMI might represent risk factors leading to worsened glycemic levels. Altogether, this evidence provides some preliminary evidence to answer the first specific question of the current scoping review (i.e., *What are the protective and risk factors among adolescents with T1DM in the context of Covid-19*)*?*.

It is also worth noting that the complexity of diabetes management also necessitates continuous caregivers’ involvement in adolescents’ care in order to establish adaptive family *teamwork*, that sees the joint effort of both the adolescent and the caregiver and thereby prevent the worsening of glycemic control and the maintenance of adherence (14). Indeed, the family provides key support for the optimal management of a child’s diabetes, whereby if family conflicts occur, the adolescent’s psychophysical health might be affected as well. In this regard, the second specific question to be answered in the present scoping review is “*How did Covid-19 influence the role of caregivers in supporting their adolescents with T1DM?”*. Three studies assessed the emotional impact of Covid-19 (27, 28, 33) upon the caregivers of adolescents with T1DM, also investigating their incidence of mental health disorders as compared to parents from the general population (28). These authors showed mixed results: on the one hand, caregivers have experienced symptoms of anxiety, uncertainty and worries related to the suspension of medical appointments, along with an augmented need to provide care to their child (27, 28); on the other hand, caregivers did not show any worries specifically related to Covid-19 and their DD levels have even decreased (33). As regards this latter result, and as previously pointed out, some studies have speculated a *“positive lockdown effect”* (28, 30, 34), suggesting that a more stable routine in adolescents and caregivers resulting from the limitations imposed by the pandemic may underline the positive and favorable evidence identified regarding glycemic control and DD. Notwithstanding, given the little research identified and evaluated, the need for prevention interventions and support programs directed to all adolescents with T1DM and their caregivers should still be considered, particularly taking into account the current continuous changes in the pandemic-related restrictions, which strongly limits the possibility for stable lifestyle routines. Therefore, following this line, intervention measures represent the next step to be taken into consideration and subsequently implemented, as a mean of support and/or prevention for adolescents with T1DM and their caregiver(s).

In the context of the Covid-19 pandemic, it is even more necessary to encourage and increase, pediatric access to CGM devices, in order to favor glycemic monitoring and data sharing with healthcare professionals and the caregivers that assist the adolescent with T1DM. In this regard, it is worth mentioning that mental health professionals should be intended as active components of the multidisciplinary team, equally involved in providing psychosocial support to all caregivers and adolescents with T1DM (14). Accordingly, digital health solutions directed to both psychosocial and medical aspects should also be developed and promulgated. Indeed, the third and last specific question to be answered in the current scoping review is “*Which prevention measures have been implemented so far to support adolescents with T1DM in the context of Covid-19?”*. Only one of the included studies has investigated the role of telemedicine in monitoring adolescents’ glycemic control. The authors found an improvement in the glycemic control among adolescents *via* the continuous assistance deployed through telemedicine during and even post-lockdown, speculating a *“dragging effect”* (5, p. 5). Nonetheless, the authors themselves stated that there is no evidence to prove the efficacy of this digital intervention on adolescents’ improved glycemic levels (30). Still, the literature shows that most eHealth interventions are predominantly oriented towards adults with diabetes, and they are mainly pilot or proof-of-concept studies (36–38).

## Limitations

The present scoping review allowed for the delineation of a comprehensive picture of the available literature on the impact of Covid-19 upon adolescents with T1DM and their caregivers; however, this review did not provide a quantitative synthesis of effect sizes. Moreover, diabetic ketoacidosis (DKA), a complication of T1DM, was not included as an outcome in the present study for its specific characteristics, thus, being DKA a rather common and relevant complication in adolescents with T1DM. Future research is needed to consider DKA weight in diabetes management among adolescents. Lastly, only one author (GDA) independently screened the titles and abstracts. However, this phase of the search process was a preliminary step based only on the evaluation of title and abstract, while the more specific and thorough full-text screening has been conducted following a double-blind process.

## Conclusions

The main and broader question of the present scoping review was: *“How has Covid-19 influenced diabetes management and well-being among adolescents with T1DM and their caregivers?”*. It is interesting to note that during the lockdown period a stable routine seems to have played a protective role towards glycemic control and DD among adolescents with T1DM, to the point that the term “positive lockdown effect” was employed. Furthermore, being an older adolescent has also emerged as a possible protective factor for glycemic control, which might be given by the greater psycho-neurological maturity of older adolescents compared to younger ones. On the contrary, a higher BMI was associated with worsened glycemic levels, which stresses the need to carefully monitor eating habits as well as physical activity with prompt attention and intervention. Referring to caregivers, studies showed limited and mixed results regarding their psychological state during the Covid-19 pandemic. Altogether, preliminary findings from the included studies do not allow to draw sound conclusions regarding risk and protective measures, and thus on the actual impact of the Covid-19 upon adolescents with T1DM and their caregiver. Thereby, future research should endeavor to answer the three questions posed by this review in order to gain an in-depth understanding of the underlying risks and protective factors that Covid-19 may have raised among adolescents with T1DM and their caregivers. Moreover, interventions supporting the medical and psychological well-being of both adolescents and caregivers during the Covid-19 pandemic should be strengthened, and new and effective interventions, both in digital and in traditional settings, should be developed. A further important aspect that has emerged from the current scoping review is the need to conduct studies that more carefully distinguish between children and adolescents with T1DM, as their developmental challenges are distinct and have different implications, even more so if a chronic disease is involved. Lastly, this scoping review permitted to highlight the need to also consider psychosocial variables relevant to T1DM when investigating the impact of Covid-19, as the psychosocial components of the disease are influenced by the medical ones and vice versa. Indeed, both psychosocial and medical variables are bidirectionally associated (39) and they are comparably relevant for the investigation, prevention, and promotion of the broader well-being of adolescents with T1DM. In addition, the joint consideration of the two may be of practical benefit in better comprehending the whole functioning of adolescents with T1DM. This would allow to further tailor interventions upon the specific characteristics of this age group, which would be of great value particularly during the pandemic period.

## Author Contributions

GB and EM defined the search strategy. GD’A did the first article screening. GB and EM did the full-text screening and their selection. GB and GD’A did the data extraction. EM conducted the synthesis and GB wrote the narrative summary. SS critically revised the summary and manuscript for important intellectual content. All authors contributed to the article and approved the submitted version.

## Conflict of Interest

The authors declare that the research was conducted in the absence of any commercial or financial relationships that could be construed as a potential conflict of interest.

## Publisher’s Note

All claims expressed in this article are solely those of the authors and do not necessarily represent those of their affiliated organizations, or those of the publisher, the editors and the reviewers. Any product that may be evaluated in this article, or claim that may be made by its manufacturer, is not guaranteed or endorsed by the publisher.
