# Supplementary material for: The Impact of the Covid-19 Pandemic on the Well-Being and Diabetes Management of Adolescents With Type 1 Diabetes and Their Caregivers: A Scoping Review
Source: Front Clin Diabetes Healthc. 2022 Mar 8;3:835598. doi: 10.3389/fcdhc.2022.835598 (PMC10012096; doi:10.3389/fcdhc.2022.835598)
Supplement: Supplementary file 1 [file Table_1.docx]

Supplementary Material

# Table S1. Excluded studies with reason

| **Authors, year** | **Reasons for exclusion** |
| --- | --- |
| Al Agha, 2021 (1) | The study’s population did not meet our age range criterion |
| Alharthi, 2021 (2) | The study’s population did not meet our age range criterion |
| Caruso, 2021 (3) | The study’s population did not meet our age range criterion |
| Cheng, 2021 (4) | The study’s population did not meet our age range criterion |
| Christoforidis, 2020 (5) | The study’s population did not meet our age range criterion |
| Cognigni, 2021 (6) | The study’s population did not meet our age range criterion |
| Cusinato, 2021 (7) | The study’s population did not meet our age range criterion |
| D'Annunzio, 2020 (8) | The study’s population did not meet our age range criterion |
| Di Riso, 2021 (9) | The study’s population did not meet our age range criterion |
| Dilek, 2021 (10) | The study’s population did not meet our age range criterion; included patients with Diabetic Ketoacidosis (DKA) |
| Gómez, 2021 (11) | The study’s population did not meet our age range criterion |
| Grabia, 2020 (12) | The study’s population did not meet our age range criterion |
| Güemes, 2020 (13) | The study’s population did not meet our age range criterion |
| Klatman, 2020 (14) | The study did not comply with our review’s aim (i.e., provided a broad overview of Diabetes during the Covid-19 pandemic) |
| Krisiunas, 2020 (15) | The study did not comply with our review’s aim (i.e., focused on mHealth during the Covid-19 pandemic) |
| Lim, 2020 (16) | The study’s population did not meet our age range criterion |
| Ludvigsson, 2021 (17) | The study’s population did not meet our age range criterion |
| McGlacken-Byrne, 2021 (18) | The study’s population did not meet our age range criterion; the study did not comply with our review’s aim (i.e., focused on severity of presentation of childhood onset T1DM during the Covid-19 pandemic) |
| Nwosu, 2021 (19) | The study’s population did not meet our age range criterion |
| NR, 2020 (20) | No full-text available |
| NR, 2020 (21) | The article’s full-text was available in Chinese only |
| Rabbone, 2020 (22) | The study’s population did not meet our age range criterion |
| Rachmiel, 2021 (23) | The study’s population did not meet our age range criterion |
| Salabelle, 2021 (24) | The study’s population did not meet our age range criterion |
| Sánchez Conejero, 2021 (25) | The study’s population did not meet our age range criterion; full-text was available in Spanish only |
| Scaramuzza, 2021 (26) | The study’s population did not meet our age range criterion; the study did not comply with our review’s aim (i.e., just provided recommendations against seasonal flu for children, adolescents and adults with T1DM during the Covid-19 pandemic) |
| Schiaffini, 2020 (27) | The study’s population did not meet our age range criterion |
| Scott, 2021 (28) | The study’s population did not meet our age range criterion |
| Shah, 2021 (29) | The study’s population did not meet our age range criterion |
| Tejera-Perez, 2021 (30) | The study’s population did not meet our age range criterion and included both patients with T1DM and T2DM. |
| Tittel, 2020 (31) | The study did not comply with our review’s aim (i.e., focused on the incidence of Diabetes during the Covid-19 pandemic) |
| Tornese, 2020 (32) | The study’s population did not meet our age range criterion |
| Verma, 2020 (33) | The study’s population did not meet our age range criterion and included patients with Diabetic Ketoacidosis (DKA) |
| Verma, 2021 (34) | The study’s population did not meet our age range criterion |
| Vigersky, 2021 (35) | The study’s population did not meet our age range criterion; the study did not comply with our review’s aim (i.e., focused on mHealth during the Covid-19 pandemic) |
| Vlad, 2021 (36) | The study’s population did not meet our age range criterion; the study did not comply with our review’s aim (i.e., focused on the incidence of Diabetes during the Covid-19 pandemic) |
| Zubkiewicz-Kucharska, 2021 (37) | The study’s population did not meet our age range criterion; the study did not comply with our review’s aim (i.e., focused on the incidence of Diabetes during the Covid-19 pandemic) |

**References:**

1. Al Agha AE, Alharbi RS, Almohammadi OA, Yousef SY, Sulimani AE, Alaama RA. Impact of COVID-19 lockdown on glycemic control in children and adolescents. SAUDI Med J. 2021;42(1):44–8.

2. Alharthi SK, Alyusuf EY, Alguwaihes AM, Alfadda A, Al-Sofiani ME. The impact of a prolonged lockdown and use of telemedicine on glycemic control in people with type 1 diabetes during the COVID-19 outbreak in Saudi Arabia. Diabetes Res Clin Pract. 2021 Mar 1;173.

3. Caruso I, Di Molfetta S, Guarini F, Giordano F, Cignarelli A, Natalicchio A, et al. Reduction of hypoglycaemia, lifestyle modifications and psychological distress during lockdown following SARS-CoV-2 outbreak in type 1 diabetes. Diabetes Metab Res Rev. 2021;37(6).

4. Cheng HP, Wong JSL, Selveindran NM, Hong JYH. Impact of COVID-19 lockdown on glycaemic control and lifestyle changes in children and adolescents with type 1 and type 2 diabetes mellitus. Endocrine. 2021 Sep 1;73(3):499–506.

5. Christoforidis A, Kavoura E, Nemtsa A, Pappa K, Dimitriadou M. Coronavirus lockdown effect on type 1 diabetes management on children wearing insulin pump equipped with continuous glucose monitoring system. DIABETES Res Clin Pract. 2020 Aug 1;166.

6. Cognigni M, D’Agostin M, Schiulaz I, Giangreco M, Carletti C, Faleschini E, et al. HbA1c and BMI after lockdown for COVID-19 in children and adolescents with type 1 diabetes mellitus. ACTA Paediatr. 2021;110(7):2206–7.

7. Cusinato M, Martino M, Sartori A, Gabrielli C, Tassara L, Debertolis G, et al. Anxiety, depression, and glycemic control during Covid-19 pandemic in youths with type 1 diabetes. J Pediatr Endocrinol Metab. 2021;34(9):1089–93.

8. d’Annunzio G, Maffeis C, Cherubini V, Rabbone I, Scaramuzza A, Schiaffini R, et al. Caring for children and adolescents with type 1 diabetes mellitus: Italian Society for Pediatric Endocrinology and Diabetology (ISPED) statements during COVID-19 pandemia. DIABETES Res Clin Pract. 2020;168.

9. Di Riso D, Bertini S, Spaggiari S, Olivieri F, Zaffani S, Comerlati L, et al. Short-Term Effects of COVID-19 Lockdown in Italian Children and Adolescents with Type 1 Diabetes Mellitus: The Role of Separation Anxiety. Int J Environ Res Public Health. 2021 Jun 1;18(11).

10. Dilek SÖ, Gürbüz F, Turan H, Celiloǧlu C, Yüksel B. Changes in the presentation of newly diagnosed type 1 diabetes in children during the COVID-19 pandemic in a tertiary center in Southern Turkey. J Pediatr Endocrinol Metab. 2021;

11. Gómez AM, Henao D, Parra D, Kerguelen A, Pinilla MV, Muñoz OM, et al. Virtual training on the hybrid close loop system in people with type 1 diabetes (T1D) during the COVID-19 pandemic. Diabetes Metab Syndr Clin Res Rev. 2021 Jan 1;15(1):243–7.

12. Grabia M, Markiewicz-żukowska R, Puścion-Jakubik A, Bielecka J, Nowakowski P, Gromkowska-Kępka K, et al. The nutritional and health effects of the COVID-19 pandemic on patients with diabetes mellitus. Nutrients. 2020;12(10):1–15.

13. Güemes M, Storch-De-Gracia P, Enriquez SV, Martín-Rivada Á, Brabin AG, Argente J. Severity in pediatric type 1 diabetes mellitus debut during the COVID-19 pandemic. J Pediatr Endocrinol Metab. 2020 Dec 1;33(12):1601–3.

14. Klatman EL, Besançon S, Bahendeka S, Mayige M, Ogle GD. COVID-19 and type 1 diabetes: Challenges and actions. Diabetes Res Clin Pract. 2020 Aug 1;166.

15. Krisiunas E, Sibomana L. Benefits of Technology in the Age of COVID-19 and Diabetes..Mobile Phones From a Rwanda Perspective. J Diabetes Sci Technol. 2020;14(4):748–9.

16. Lim ST, Yap F, Chin X. Bridging the Needs of Adolescent Diabetes Care During COVID-19: A Nurse-Led Telehealth Initiative. J Adolesc Heal. 2020 Oct 1;67(4):615–7.

17. Ludvigsson J. Effect of COVID-19 pandemic on treatment of Type 1 diabetes in children. Acta Paediatr Int J Paediatr. 2021 Mar;110(3):933–4.

18. McGlacken-Byrne SM, Drew SE V, Turner K, Peters C, Amin R. The SARS-CoV-2 pandemic is associated with increased severity of presentation of childhood onset type 1 diabetes mellitus: A multi-centre study of the first COVID-19 wave. Diabet Med. 2021;38(9).

19. Nwosu BU, Al-Halbouni L, Parajuli S, Jasmin G, Zitek-Morrison E, Barton BA. COVID-19 Pandemic and Pediatric Type 1 Diabetes: No Significant Change in Glycemic Control During The Pandemic Lockdown of 2020. Front Endocrinol (Lausanne). 2021;12.

20. [Anonymous]. Corona Pandemic apparently causes delayed Diagnosis of Type 1 Diabetes Mellitus in Children and Adolescents. MONATSSCHRIFT Kinderheilkd. 2020 Nov;168(11, SI):986.

21. [Anonymous]. The Corona Pandemic is apparently causing the belated Diagnosis of Type 1 Diabetes mellitus in Children and Adolescents. DIABETOLOGE. 2020;16(6, SI):583.

22. Rabbone I, Schiaffini R, Cherubini V, Maffeis C, Scaramuzza A. Has covid-19 delayed the diagnosis and worsened the presentation of type 1 diabetes in children? Diabetes Care. 2020 Nov;43(11):2870–2.

23. Rachmiel M, Lebenthal Y, Mazor-Aronovitch K, Brener A, Levek N, Levran N, et al. Glycaemic control in the paediatric and young adult population with type 1 diabetes following a single telehealth visit - what have we learned from the COVID-19 lockdown? Acta Diabetol. 2021;58(6):697–705.

24. Salabelle C, Ly Sall K, Eroukhmanoff J, Franc S, Oumbiche H, Zrafi WS, et al. COVID-19 pandemic lockdown in young people with type 1 diabetes: Positive results of an unprecedented challenge for patients through telemedicine and change in use of continuous glucose monitoring. Prim Care Diabetes. 2021;15(5):884–6.

25. Sánchez Conejero M, González de Buitrago Amigo J, Tejado Bravo ML, de Nicolás Jiménez JM. Impact of COVID-19 lockdown on glucemic control in children and adolescents with type 1 diabetes mellitus [Repercusión del confinamiento por COVID-19 sobre el control glucémico en ni˜nos y adolescentes con diabetes mellitus tipo 1]. An Pediatr. 2021;

26. Scaramuzza AE, Rabbone I, Maffeis C, Schiaffini R. Seasonal flu and COVID-19 recommendations for children, adolescents and young adults with diabetes. Diabet Med. 2021;38(1).

27. Schiaffini R, Barbetti F, Rapini N, Inzaghi E, Deodati A, Patera IP, et al. School and pre-school children with type 1 diabetes during Covid-19 quarantine: The synergic effect of parental care and technology. Diabetes Res Clin Pract. 2020 Aug 1;166.

28. Scott SN, Fontana FY, Züger T, Laimer M, Stettler C. Use and perception of telemedicine in people with type 1 diabetes during the COVID-19 pandemic—Results of a global survey. Endocrinol Diabetes Metab. 2021;4(1).

29. Shah N, Karguppikar M, Bhor S, Ladkat D, Khadilkar V, Khadilkar A. Impact of lockdown for COVID-19 pandemic in Indian children and youth with type 1 diabetes from different socio-economic classes. J Pediatr Endocrinol Metab. 2021 Feb 1;34(2):217–23.

30. Tejera- Perez C, Moreno-Pérez Ó, Rios J, Reyes-García R. People living with type 1 diabetes point of view in COVID-19 times (COVIDT1 study): Disease impact, health system pitfalls and lessons for the future. Diabetes Res Clin Pract. 2021 Jan 1;171.

31. Tittel SR, Rosenbauer J, Kamrath C, Ziegler J, Reschke F, Hammersen J, et al. Did the COVID-19 lockdown affect the incidence of pediatric type 1 diabetes in Germany? Diabetes Care. 2020 Nov;43(11):e172--e173.

32. Tornese G, Ceconi V, Monasta L, Carletti C, Faleschini E, Barbi E. Glycemic Control in Type 1 Diabetes Mellitus During COVID-19 Quarantine and the Role of In-Home Physical Activity. DIABETES Technol \& Ther. 2020;22(6):462–7.

33. Verma A, Rajput R, Verma S, Balania VKB, Jangra B. Impact of lockdown in COVID 19 on glycemic control in patients with type 1 Diabetes Mellitus. Diabetes Metab Syndr Clin Res Rev. 2020 Sep 1;1213–6.

34. Verma A, Verma S, Dochania K, Vaswani N Das. Effect of COVID 19 Second Wave on Children with type 1 Diabetes Mellitus in India. Diabetes Metab Syndr. 2021;15(4):102171.

35. Vigersky RA, Velado K, Zhong A, Agrawal P, Cordero TL. The Effectiveness of Virtual Training on the MiniMed^TM^ 670G System in People with Type 1 Diabetes during the COVID-19 Pandemic. Diabetes Technol Ther. 2021 Feb;23(2):104–9.

36. Vlad A, Serban V, Timar R, Sima A, Botea V, Albai O, et al. Increased Incidence of Type 1 Diabetes during the COVID-19 Pandemic in Romanian Children. MEDICINA-LITHUANIA. 2021 Sep 1;57(9).

37. Zubkiewicz-Kucharska A, Seifert M, Stępkowski M, Noczyńska A, Stepkowski M, Noczynska A. Diagnosis of type 1 diabetes during the SARS-CoV-2 pandemic: Does lockdown affect the incidence and clinical status of patients. Adv Clin Exp Med. 2021 Feb 1;30(2):127–34.
